# Diversity, natural infection and blood meal sources of phlebotomine sandflies (Diptera, Psychodidae) in the western Brazilian Amazon

**DOI:** 10.1590/0074-02760190170

**Published:** 2019-07-29

**Authors:** Antonio Marques Pereira, Ana Beatriz Nascimento Souza, Thaís Santos Castro, Michelli Santos da Silva, Paula Frassinetti Medeiros de Paulo, Gabriel Eduardo Melim Ferreira, Jansen Fernandes de Medeiros

**Affiliations:** 1 Fundação Universidade Federal de Rondônia, Programa de Pós-Graduação em Biologia Experimental, Porto Velho, RO, Brasil; 2 Fundação Oswaldo Cruz, Laboratório de Entomologia, Porto Velho, RO, Brasil; 3 Instituto Nacional de Epidemiologia na Amazônia Ocidental, Porto Velho, RO, Brasil; 4 Fundação Oswaldo Cruz, Laboratório de Epidemiologia Genética, Porto Velho, RO, Brasil

**Keywords:** Leishmania, vector, reservoirs, protected areas, state of Rondônia

## Abstract

**BACKGROUND:**

The state of Rondônia (RO) is a hot spot for human cases of cutaneous leishmaniasis. Many sandfly species in RO are putative vectors of leishmaniasis.

**OBJECTIVES:**

This study examines the diversity patterns and the presence of *Leishmania* DNA and blood meal sources of sandflies in RO.

**METHODS:**

A sandfly survey was performed between 2016 and 2018 in 10 municipalities categorised into three different environment types: (i) Conservation Unit (CUN) - comprised of preserved ombrophilous forests; (ii) Forest Edge (FE) - small forest fragments; and (iii) Peridomicile (PE) - areas around dwellings.

**FINDINGS:**

A total of 73 species were identified from 9,535 sandflies. The most abundant species were *Psychodopygus davisi* (1,741 individuals), *Nyssomyia antunesi* (1,397), *Trichophoromyia auraensis* (1,295) and *Trichophoromyia ubiquitalis* (1,043). Diversity was the highest in CUN, followed by the FE and PE environments. One pool of *Ps*. *davisi* tested positive for *Leishmania braziliensis*, reinforcing the possibility that *Ps*. *davisi* acts as a vector. The cytochrome b (*cyt*b) sequences were used to identify three blood meal sources: *Bos taurus*, *Homo sapiens* and *Tamandua tetradactyla*.

**MAIN CONCLUSIONS:**

Our results demonstrated that sandflies can switch between blood meal sources in differing environments. This study enhances the knowledge of the vector life cycle in RO and provides information relevant to leishmaniasis surveillance.

Phlebotomine sandflies (Diptera, Psychodidae) are small insects that act as natural vectors for protozoans of the genus *Leishmania* Ross (Kinetoplastida, Trypanosomatidae), which causes cutaneous leishmaniasis (CL) and visceral leishmaniasis (VL) in humans.^1^ Sandflies are distributed worldwide, but their diversity is the highest in the neotropical region, where 530 species have been recorded.^2^

Of the 280 sandfly species found in Brazil, 13 species are the proven vectors of leishmaniasis^3^ and 11 species, including *Bichromomyia flaviscutellata* (Mangabeira), *Bichromomyia olmeca nociva* (Young & Arias), *Migonemyia migonei* (França), *Nyssomyia anduzei* (Rozeboom), *Nyssomyia antunesi* (Coutinho), *Nyssomyia umbratilis* (Ward & Fraiha), *Nyssomyia whitmani* (Antunes & Coutinho), *Psychodopygus complexus* (Mangabeira), *Psychodopygus davisi* (Root), *Psychodopygus wellcomei* (Fraiha, Shaw & Lainson), and *Trichophoromyia ubiquitalis* (Mangabeira), might be involved in the transmission of *Leishmania* that cause CL.^3^

Brazil is also home to a range of putative vectors such as *Nyssomyia intermedia*, *Nyssomyia neivai*,^3^ *Nyssomyia shawi*,^4^ *Psychodopygus carrerai*, *Psychodopygus hirsutus hirsutus* ^5^ ^-^ ^7^ and *Trichophoromyia auraensis* (Mangabeira). *Leishmania* (*Leishmania*) *infantum* Nicolle is the causative agent of VL, and its primary vector is *Lutzomyia longipalpis* (Lutz & Neiva), which is distributed throughout Brazil.^2^ Other species associated with the transmission of *Le*. (*Le*.) *infantum* include *Lutzomyia cruzi* (Man-gabeira),^8^ *Mg*. *migonei* ^9^ and *Pintomyia fischeri*.^10^

CL occurs in all regions of the Brazilian Amazon. Its incidence rates are the highest in the states of Pará (PA) and Amazonas (AM).^11^ In contrast, VL is endemic to the states of Mato Grosso (MT), Mato Grosso do Sul, Roraima, Tocantins and PA.^11^ The zoonotic transmission risk and incidence of this disease are increased by certain factors, including (i) the presence of an ombrophilous forest, (ii) a variety of blood meal sources (e.g., tapirs, opossums, sloths and armadillos), (iii) a variety of *Leishmania* species and (iv) the presence of sandfly vector species.^5^ ^,^ ^12^ ^,^ ^13^ In major transmission foci, high incidences of CL might also be the result of human exposure to the environment through activities, such as hunting and fishing, or the result of recent deforestation caused by the construction of hydroelectric power plants and roads.^14^

RO is located in the western Amazon basin; it borders the states of Acre (AC), AM and MT and it shares an international frontier with Bolivia. The state is comprised of open ombrophilous forests that have been decreasing in area since the 1970s as a result of agriculture activities. RO has the third highest incidence of CL in north Brazil, having registered approximately 12,000 cases between 2007 and 2018; according to the Brazilian Health Ministry, this incidence rate is the result of intense zoonotic transmission.^11^ No human cases of VL have been reported in RO; however, this state is thought to be at risk for VL because canine cases have recently occurred in the municipality of Cacoal.^15^ Previous studies have reported that human cases of CL in RO have been caused by the following *Leishmania* species: *Leishmania* (*Leishmania*) *amazonensis* Lainson & Shaw, *Leishmania* (*Viannia*) *braziliensis* Vianna, *Leishmania* (*Viannia*) *guyanensis* Floch, *Leishmania* (*Viannia*) *lainsoni* Silveira, Ishikawa, Souza & Lainson, *Leishmania* (*Viannia*) *lindenbergi* Silveira, Ishikawa & Sousa, and *Leishmania* (*Viannia*) *shawi* Lainson, Braga, Souza, Póvoa & Ishikawa.^16^

Despite the presence and high diversity of known CL vectors, such as *Bi*. *flaviscutellata*, *Ny*. *antunesi*, *Ny. umbratilis* and *Th*. *ubiquitalis*,^4^ ^,^ ^13^ few studies conducted in RO have demonstrated the natural infection of sandflies.^4^ ^,^ ^7^ ^,^ ^13^ ^,^ ^17^ The presence and high abundance of *Leishmania* in RO suggest that sandflies maintain their transmission cycles in the this region.^4^ ^,^ ^7^ ^,^ ^13^ ^,^ ^17^

The epidemiological knowledge of sandflies can be improved by studying their blood meal sources^18^ ^,^ ^19^ and identifying putative reservoirs. The mitochondrial genes, such as cytochrome *b* (*cyt*b), have been used as molecular markers for the detection of their blood meal sources.^18^ ^-^ ^20^ Thus, the present study aimed to characterise the sandfly fauna and identify their blood meal sources, as well as to assess the natural infection caused by *Leishmania* in RO.

## MATERIALS AND METHODS

**Fig. 1:**
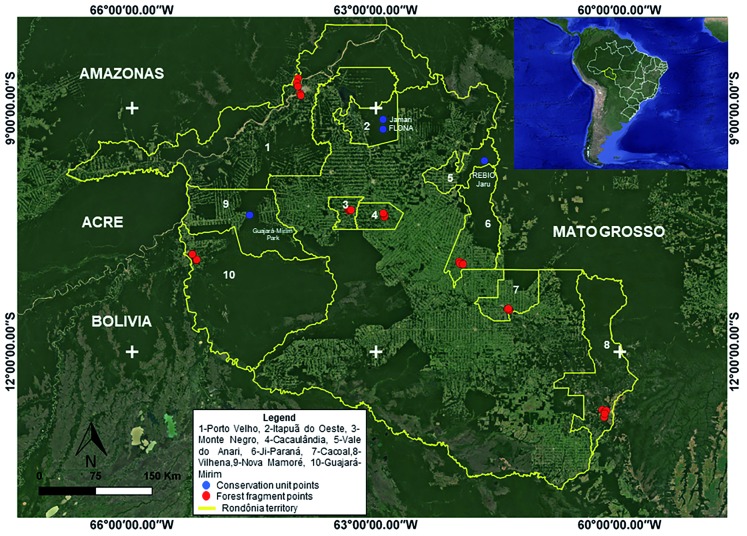
sandfly collection points distributed in the state of Rondônia, Brazil.

*Study areas -* RO is located in the northern region of Brazil; it borders AM to the north, MT to the east and AC to the west and shares an international border with Bolivia to the southwest (Fig. 1). It has an area of approximately 238,000 km² and contains 52 municipalities.

The sandfly fauna was collected in three environments: Conservation Unit (CUN) - characterised by large areas of ombrophilous rain forest; Forest Edge (FE) - characterised by small forest fragments near urban areas; and Peridomicile (PE) - areas around dwellings that are situated near small forest fragments and contain enclosures where domestic animals are raised. Collections in the CUN environment were conducted between 2016 and 2017 at three places (Fig. 1): the Jaru Biological Reserve (REBIO Jaru), which has a territory that covers six municipalities (Machadinho D’Oeste, Vale do Anari, Theobroma, Ouro Preto do Oeste, Vale do Paraíso and Ji-Paraná) where collection were made in May and December of 2016 and April and July of 2017; the Jamari National Forest (FLONA Jamari), located north of RO in the municipality of Itapuã do Oeste, where collections were made in April and August of 2016 and April and October of 2017; and Guajará-Mirim State Park, located to the west of RO between the municipalities of Nova Mamoré and Guajará-Mirim, where collections were made in May and August of 2016 and April and November of 2017.

Collections in the FE and PE environments were conducted between 2016 and 2018 in the municipalities of Cacaulândia and Monte Negro (where collections were made in October 2016, June 2017 and May 2018); Cacoal, Ji-Paraná and Vilhena (where collections were made in July 2016, May 2017 and April 2018); and Guajará-Mirim and Porto Velho (where collections were made in May 2016, April 2017, and June 2018) (Fig. 1).

*Sandfly collection and identification -* In the CUN environments, collections were made along two different trails and sampling was performed twice in 2016 and twice in 2017. Six Hoover Pugedo (HP) light traps were set along each trail (12 HPs/reserve) and collections were made between 06:00 p.m. and 07:00 a.m. for five consecutive days.

Collections in the FE and PE environments were made from 2016 to 2018 at five locations within each municipality. At each location, one trap was set in the FE environment and two traps were set in the PE environment, using a total of 15 traps per municipality.

Male sandflies were clarified in 10% potassium hydroxide (KOH), washed in 10% acetic acid and slide-mounted in Berlese fluid. Females were divided into engorged and non-engorged specimens, and their heads and genitalia were clarified and slide-mounted as above. The thorax and abdomen of each female were stored in a microtube with 96% ethylic alcohol for further molecular analysis. Species identification was carried out using the morphological characters described by Galati.^2^

*Molecular detection of Leishmania -* Females were sorted according to species abundance, collection location and environment type and were separated into pools of 2-20 specimens. DNA extraction and polymerase chain reaction (PCR) assays were performed by targeting *k*DNA and hsp70, as described elsewhere.^4^ ^,^ ^21^ The *Th*. *ubiquitalis* males and the *Le*. *amazonensis* reference strain IOC/L0575 (IFLA/BR/1967/PH8) were used as positive controls and ultrapure water was used as the negative control.

*Sandfly blood meal sources -* Engorged females were separated according to species, municipality, and environment type. During DNA extraction, three samples were used as negative controls: one sample containing DNA-free water and two samples containing a female sandfly with no blood present in the gut. DNA extraction was carried out using the phenol/chloroform protocol described by Sambrook and Russell.^22^ A PCR was carried out using the primers *cyt*b 1 and *cyt*b 2, which are complementary to the conserved region of the *cyt*b gene in vertebrate mitochondrial DNA.^23^

The PCR amplification was carried out in a 50 µL reaction volume containing 25 µL (1X) Go Taq Colorless (Promega^®^, Madison, WI, USA), 1.5 µL of each primer (*cyt*b 1 and *cyt*b 2, 10 µM each) and 5 µL of DNA (< 250 ng). The amplifications were performed in a thermocycler (Veriti^®^ - Applied Biosystems, Foster City, CA, USA) with an initial denaturation of 95ºC for 5 min, followed by 35 cycles of denaturation at 95ºC for 30 s, annealing at 53ºC for 30 s and extension at 72ºC for 1 min, with a final extension at 72ºC for 6 min. Amplified products were purified using the QIAquick Purification Kit (Qiagen, Hilden, Germany) and submitted to the Fiocruz Sequencing Facility (Rio de Janeiro, RJ, Brazil).

*Data analysis -* Interpolation and extrapolation curves (iNEXT) were used to evaluate sample coverage and compare diversity indexes (Shannon and Simpson) between environments. Comparisons were made using Hill numbers expressed as order q values, and the data were analysed in the R program.^7^

The sequences (hsp 70 and *cyt*b) were analysed using the Phred, Phrap and Consed software programs,^24^ with the minimum value defined as Q = 30. The consensus sequences were submitted to the Basic Local Alignment Search Tool (BLAST) (http://blast.ncbi.nlm.nih.gov/Blast.cgi) and compared with the sequences obtained from the National Center for Biotechnology Information (NCBI) GenBank database (http://www.ncbi.nlm.nih.gov/genbank/).

*Ethics -* The study was performed under authorisations 43702-1 and 56321-1 SISBIO/ICMBio/MMA.

## RESULTS

A total of 73 species and 14 genera were identified from 9,535 individuals (♀4,089/♂5,446) (Table I). Owing to the absence of morphological characters, 118 individuals were identified only at the genus level, with 49 individuals belonging to the genus *Trichophoromyia* and 69 to *Trichopygomyia*. The most abundant species were *Ps*. *davisi* (1,741 individuals), *Ny*. *antunesi* (1,397), *Th*. *auraensis* (1,295) and *Th*. *ubiquitalis* (1,043); these four species comprised 57% of all individuals collected.

A sample coverage analysis indicated that sandfly populations were sufficiently represented in all environments. The CUN, FE and PE environments yielded 5,847, 2,111 and 1,457 individuals and 68, 58 and 47 species, respectively, at 99% sample coverage (Table I).

**TABLE I:** Sandfly composition, exponential of Shannon entropy index (q = 1) and inverse of Simpson concentration index (q = 2) with its confidence intervals (CI) based on a bootstrap method of 1,000 replications for three environments from the state of Rondônia, Brazil

Species	CUN	FE	PE	Total	%
	Total (♀/♂)						
*Bichromomyia flaviscutellata* (Mangabeira, 1942)^*a*^	65 (40/25)	65 (23/42)	9 (6/3)	139	1.46
*Bichromomyia olmeca nociva* (Young & Arias, 1970)	-	-	1 (1/0)	1	0.01
*Brumptomyia brumpti* (Larrousse, 1920)^*a*^	14 (2/12)	9 (5/4)	10 (3/7)	33	0.35
*Brumptomyia mesai* Sherlock, 1962	1 (0/1)	-	-	1	0.01
*Brumptomyia pintoi* (Costa Lima, 1932)	1 (0/1)	-	-	1	0.01
*Evandromyia bacula* (Martins, Falcão & Silva, 1965)^*a*^	9 (6/3)	2 (1/1)	2 (1/1)	13	0.14
*Evandromyia georgii* (Freitas & Barrett, 2002)^*a*^	22 (19/3)	16 (12/4)	5 (3/2)	43	0.45
*Evandromyia infraspinosa* (Mangabeira, 1941)	7 (5/2)	-	-	7	0.07
*Evandromyia lenti* (Mangabeira, 1938)	-	2 (1/1)	2 (1/1)	4	0.04
*Evandromyia piperiformis* Godoy, Cunha & Galati, 2017	-	-	1 (0/1)	1	0.01
*Evandromyia saulensis* (Floch & Abonnenc, 1944)^*a*^	62 (51/11)	18 (13/5)	14 (11/3)	94	0.99
*Evandromyia tarapacaensis* (Le Pont, Torrez-Espejo & Galati, 1997)	2 (0/2)	2 (0/2)	-	4	0.04
*Evandromyia termitophila* (Martins, Falcão & Silva, 1964)^*a*^	2 (0/2)	4 (2/2)	1 (1/0)	7	0.07
*Evandromyia walkeri* (Newstead, 1941)^*a*^	29 (27/2)	4 (3/1)	46 (15/31)	79	0.83
*Evandromyia wilsoni* (Dasmasceno & Causey, 1945)	17 (4/13)	3 (1/2)	-	20	0.21
*Lutzomyia evangelistai* Martins & Fraiha, 1971	2 (0/2)	-	-	2	0.02
*Lutzomyia marinkellei* Young, 1979	2 (0/2)	-	-	2	0.02
*Lutzomyia sherlocki* Martins, Silva & Falcão, 1971^*a*^	72 (47/25)	35 (23/12)	10 (8/2)	117	1.23
*Martinsmyia waltoni* (Arias, Freitas & Barrett, 1984)	-	1 (0/1)	-	1	0.01
*Micropygomyia rorotaensis* (Floch & Abonnenc, 1944)^*a*^	11 (1/10)	6 (1/5)	5 (2/3)	22	0.23
*Micropygomyia trinidadensis* (Newstead, 1922)	-	5 (3/2)	1 (1/0)	6	0.06
*Micropygomyia villelai* (Mangabeira, 1942)^*a*^	6 (6/0)	6 (5/1)	4 (4/0)	16	0.17
*Migonemyia migonei* (França, 1920)^*a*^	8 (3/5)	1 (1/0)	1 (0/1)	10	0.10
*Nyssomyia anduzei* (Rozeboom, 1942)	26 (17/9)	1 (0/1)	-	27	0.28
*Nyssomyia antunesi* (Coutinho, 1939)^*a*^	650^*b*^ (447/203)	234^*b*^ (169/65)	513^*b*^ (235/278)	1,397	14.65
*Nyssomyia delsionatali* Galati & Galvis, 2012)^*a*^	1 (0/1)	1 (0/1)	5 (0/5)	7	0.07
*Nyssomyia richardwardi* (Ready & Fraiha, 1981)	123 (97/26)	12 (11/1)	-	135	1.42
*Nyssomyia shawi* (Fraiha, Ward & Ready, 1981)	14 (9/5)	1 (1/0)	-	15	0.16
*Nyssomyia umbratilis* (Ward & Faiha, 1977)	95 (74/21)	11 (9/2)	-	106	1.11
*Nyssomyia whitmani* (Antunes & Coutinho, 1939)^*a*^	162^*b*^ (57/105)	106^*b*^ (44/62)	2 (2/0)	270	2.83
*Nyssomyia yuilli yuilli* (Young & Porter, 1972)^*a*^	70 (69/1)	12 (12/0)	61 (25/36)	143	1.50
*Pintomyia nevesi* (Damasceno & Arouck, 1956)^*a*^	21 (18/3)	18 (16/2)	7 (3/4)	46	0.48
*Pintomyia serrana* (Damasceno & Arouck, 1949)^*a*^	6 (3/3)	13 (7/6)	2 (2/0)	21	0.22
*Pintomyia* sp.	1 (0/1)	-	-	1	0.01
*Pressatia calcarata* (Martins & Silva, 1964)	1 (0/1)	-	-	1	0.01
*Pressatia triacantha* (Mangabeira, 1942)	6 (0/6)	-	-	6	0.06
*Psathyromyia aragaoi* (Costa Lima, 1932)^*a*^	36 (16/20)	8 (4/4)	2 (1/1)	46	0.48
*Psathyromyia b*. *barretoi* (Mangabeira, 1942)	1 (0/1)	1 (1/0)	-	2	0.02
*Psathyromyia campbelli* (Damasceno, Causey & Arouck, 1945)	1 (1/0)	2 (1/1)	-	3	0.03
*Psathyromyia coutinhoi* (Mangabeira, 1942)	1 (0/1)	-	-	1	0.01
*Psathyromyia dendrophyla* (Mangabeira, 1942)^*a*^	11 (5/6)	11 (5/6)	4 (3/1)	26	0.27
*Psathyromyia dreisbachi* (Causey & Damasceno, 1945)^*a*^	15 (5/8)	12 (11/1)	18 (18/0)	43	0.45
*Psathyromyia elizabethdorvalae* Brilhante, Sábio & Galati, 2017	2 (0/2)	1 (0/1)	-	3	0.03
*Psathyromyia hermanlenti* (Martins, Silva & Falcão, 1970)^*a*^	7 (7/0)	18 (2/16)	21 (11/10)	46	0.48
*Psathyromyia lutziana* (Costa Lima, 1932)^*a*^	6 (3/3)	5 (3/2)	2 (2/0)	13	0.14
*Psathyromyia runoides* (Fairchild & Hertig, 1943)	-	6 (1/5)	9 (3/6)	15	0.16
*Psychodopygus amazonensis* (Root, 1934)^*a*^	5 (5/17)	3 (3/1)	1 (1/0)	27	0.28
*Psychodopygus ayrozai* (Barretto & Coutinho, 1940)^*a*^	10 (10/0)	4 (1/3)	2 (1/1)	16	0.17
*Psychodopygus bispinosus* (Fairchild & Hertig, 1951)	6 (6/0)	-	-	6	0.06
*Psychodopygus c*. *carrerai* (Barretto, 1946)^*a*^	376^*b*^ (103/273)	57 (26/31)	23 (8/15)	456	4.78
*Psychodopygus chagasi* (Costa Lima, 1941)^*a*^	150^*b*^ (118/32)	14 (12/2)	4 (3/1)	168	1.76
*Psychodopygus claustrei* (Root, 1934)^*a*^	114 (24/90)	42 (7/35)	9 (2/7)	165	1.73
*Psychodopygus complexus* (Mangabeira, 1941)^*a*^	240^*b*^ (165/75)	14 (0/14)	9 (0/9)	263	2.76
*Psychodopygus davisi* (Root, 1934)^*a*^	715^*b*^ (442/273)	671^*b*^ (239/432)	355^*b*^ (138/217)	1,741	18.26
*Psychodopygus geniculatus* (Mangabeira, 1941)^*a*^	121^*b*^ (108/13)	21 (15/6)	9 (4/5)	151	1.58
*Psychodopygus h*. *hirsutus* (Mangabeira, 1942)^*a*^	57 (49/8)	107^*b*^ (45/52)	38 (20/18)	202	2.12
*Psychodopygus lainsoni* Fraiha & Ward, 1974^*a*^	95 (56/39)	7 (5/2)	2 (0/2)	104	1.09
*Psychodopygus leonidasdeanei* (Fraiha, Ryan, Ward, Lainson & Shaw, 1986)	115^*b*^ (97/18)	-	-	115	1.21
*Psychodopygus llanosmartinsi* (Fraiha & Ward, 1980)^*a*^	40 (26/14)	3 (2/1)	3 (1/2)	46	0.48
*Psychodopygus paraensis* (Costa Lima, 1941)	19 (8/11)	6 (1/5)	-	25	0.26
*Psychodopygus yucumensis* (Le Pont, Caillard, Tibayrenc & Desjeux, 1986)^*a*^	4 (1/3)	-	2 (0/2)	6	0.06
*Sciopemyia fluviatilis* (floch & Abonnenc, 1944)^*a*^	3 (3/0)	3 (3/0)	1 (0/1)	7	0.07
*Sciopemyia servulolimai* (Damasceno & Causey, 1945)	10 (5/5)	7 (4/3)	-	17	0.18
*Sciopemyia sordellii* (Shannon & Del Ponte, 1927)^*a*^	94 (50/44)	20 (12/8)	14 (6/8)	128	1.34
*Trichophoromyia auraensis* (Mangabeira, 1942)^*a*^	1,261^*b*^ (9/1252)	22 (2/20)	12 (0/12)	1,295	13.58
*Trichophoromyia clitella* (Young & Pérez, 1944)^*a*^	12^*b*^ (0/122)	30 (1/29)	22 (0/22)	174	1.82
*Trichophoromyia flochi* (Abonnenc & Chassignet, 1948)^*a*^	19 (0/19)	57 (0/57)	24 (0/24)	100	1.05
*Trichophoromyia readyi* (Ryan, 1986)	4 (0/4)	-	-	4	0.04
*Trichophoromyia ruifreitasi* Oliveira, Teles, Medeiros, Camargo & Pessoa, 2016	2 (0/2)	-	-	2	0.02
*Trichophoromyia* sp.	49 (48/1)	-	-	49	0.51
*Trichophoromyia ubiquitalis* (Mangabeira, 1942)^*a*^	525^*b*^ (62/463)	352^*b*^ (93/259)	166^*b*^ (35/131)	1.043	10.94
*Trichopygomyia dasypodogeton* (Castro, 1939)^*a*^	107 (1/106)	3 (0/3)	2 (0/2)	112	1.17
*Trichopygomyia rondoniensis* (Martins, Falcão & Silva)	6 (0/6)	1 (0/1)	-	7	0.07
*Trichopygomyia* sp.	69 (69/0)	-	-	69	0.72
*Viannamyia caprina* (Osorno-Mesa, Moralez & Osorno, 1972)	11 (11/0)	12 (9/3)	-	23	0.24
*Viannamyia tuberculata* (Mangabeira, 1941)^*a*^	15 (15/0)	2 (2/0)	1 (1/0)	18	0.19
Total	5,967 (2,530/3,437)	2,111 (973/1,238)	1,457 (586/871)	9,535	100
Sample coverage (%)	99	99	99	-	-
Exponential of Shannon entropy index (CI95%)	16.6 (16.5-17.7)	16.3 (15.9-17.4)	8.3 (8.0-8.3)	-	-
Inverse of Simpson concentration index (CI95%)	8.2 (8.2-8.7)	8.5 (8.5-9.3)	3.6 (3.6-4.0)	-	-

*a*: species present in all environments evaluated; *b*: abundant species in the environment. CUN: Conservation unit; FE: Forest Edge; PE: Peridomicile.

**Fig. 2:**
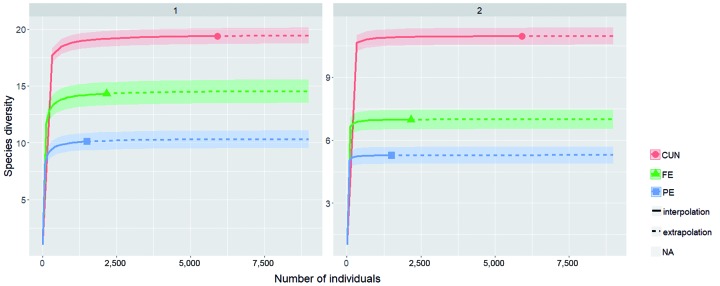
index diversities based on Hill numbers of the sandfly fauna collected in three environments in the state of Rondônia, Brazil. CUN: Conservation Unit; FE: Forest Edge; PE: Peridomicile.

Species diversity was the highest in the CUN environments, followed by the FE and PE environments. The CUN environments yielded the highest Shannon index (H’) = 19.5 common species and Simpson index (1/D) = 6.9 dominant species, followed by FE with H’ = 14.5 common species and 1/D = 6.9 dominant species and PE with H’ = 10.3 common species and 1/D = 5.2 dominant species (Table I, Fig. 2).

**Fig. 3:**
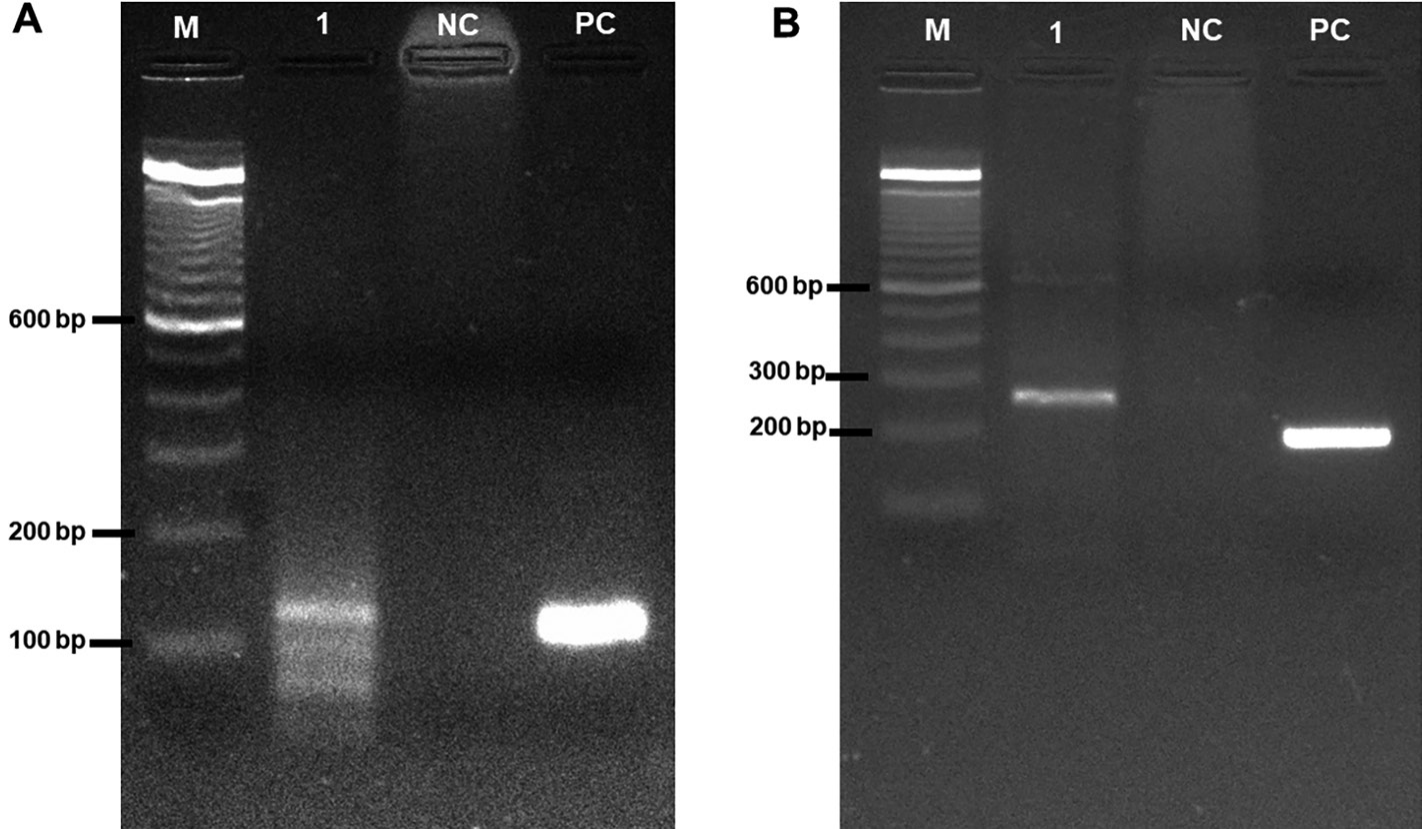
natural infection of sandfly. A: amplified fragment of 120 bp from the *k*DNA region of the kinetoplast *Leishmania* species; B: the DNA extracted from the *Psychodopygus davisi* blood sample was subjected to polymerase chain reaction (PCR), which led to the amplification of a 240 bp hsp70 fragment. The PCR products were subjected to 1.5% agarose gel electrophoresis and stained with 1 μL of GelRed^®^ solution. 1: *Ps. davisi* sample; M: molecular Maker; NC: negative control (water); PC: positive control *Leishmania amazonensis* reference strain IOC L0575 (IFLA/BR/1967/PH8).

A total of 1,755 females were divided into 274 pools representing 35 species. The PCR targeting of *k*DNA and hsp70 identified one pool of *Ps*. *davisi* infected with *Le.* (*Vi*.) *braziliensis* (query cover = 100%, identity = 100%, GenBank accession KX573933.1) (Fig. 3). The infected pool was collected from an FE environment in the municipality of Monte Negro.

Blood meal sources were identified by taking samples from 15 engorged females belonging to the following species: *Bi*. *flaviscutellata* (1), *Ny*. *antunesi* (4), *Psathyromyia dendrophyla* (2), *Ps. carrerai carrerai* (1), *Ps*. *davisi* (6) and *Ps*. *hirsutus hirsutus* (1). The samples were used to amplify a 358 bp fragment of the *cyt*b gene. The resultant sequences were compared with the GenBank sequences, leading to the identification of three vertebrates with 98-100% similarity: *Bos taurus*, *Homo sapiens* and *Tamandua tetradactyla* (Table II).

**TABLE II:** Vertebrate species identified from engorged sandfly females collected Forest Edge (FE) and Peridomicile (PE) environments in the state of Rondônia, Brazil

Sandfly species	Blood meal	Municipality (environment)	Accession code	Identity (%)	Total score (n)	Query cover (%)	E-value
*Nyssomyia antunesi*	*Tamandua tetradactyla*	Porto Velho (FE)	KT818552.1	98.14	562	100	3E-156
*Nyssomyia antunesi*	*Tamandua tetradactyla*	Porto Velho (FE)	KT818552.1	99.07	582	100	2E-162
*Nyssomyia antunesi*	*Homo sapiens*	Vilhena (PE)	KX697544.1	100	608	100	4E-170
*Bichromomyia flaviscutellata*	*Homo sapiens*	Vilhena (FE)	KX697544.1	100	643	100	1E-180
*Psathyromyia dendrophyla*	*Bos taurus*	Cacoal (PE)	MK028750.1	100	603	100	2E-168
*Psychodopygus davisi*	*Bos taurus*	Cacoal (PE)	MK028750.1	99.66	538	100	4E-149
*Psychodopygus davisi*	*Bos taurus*	Cacoal (PE)	MK028750.1	100	597	100	7E-167
*Psychodopygus davisi*	*Bos taurus*	Cacoal (PE)	MK028750.1	100	595	100	3E-166
*Psathyromyia dendrophyla*	*Bos taurus*	Cacoal (PE)	MK028750.1	99.62	481	100	6E-132
*Psychodopygus davisi*	*Bos taurus*	Cacoal (PE)	EU365345.1	99.42	625	100	4E-175
*Psychodopygus davisi*	*Bos taurus*	Cacoal (PE)	MK028750.1	100	603	100	2E-168
*Psychodopygus h. hirsutus*	*Bos taurus*	Cacoal (PE)	MK028750.1	99.66	542	100	3E-150
*Psychodopygus c. carrerai*	*Bos taurus*	Cacoal (FE)	EU365345.1	99.05	566	100	2E-157
*Psychodopygus davisi*	*Homo sapiens*	Ji-Paraná (FE)	KX697544.1	100	647	100	0.0
*Nyssomyia antunesi*	*Homo sapiens*	Ji-Paraná (PE)	KX697544.1	100	425	100	3E-115

## DISCUSSION

The epidemiological pattern of leishmaniasis in RO is characterised by a zoonotic or sylvatic transmission cycle in which humans might acquire infection via exposure to sandflies in the Amazon rainforest.^5^ ^,^ ^13^ ^,^ ^16^

A total of 73 species were registered in this study, which demonstrated a higher level of species richness than the previous surveys conducted in RO.^7^ ^,^ ^13^ ^,^ ^25^ Sandfly diversity was the highest in protected environments. The CUN environment exhibited the highest levels of species richness was found in the CUN environment, followed by the FE and PE environments; these findings corroborate those of the previous studies conducted in the Amazon region.^4^ ^,^ ^21^ ^,^ ^26^ ^,^ ^27^ Although sampling methods differed between environments, it was still possible to perform reliable diversity comparisons because the sample coverage was 99% in each environment.

Our data demonstrated that sandflies might serve as biodiversity indicators. The species richness was reduced by 10 species in the FE environments and by 21 species in the PE environments relative to the CUN environments. This indicates that the reduction of forests to small fragments affects sandfly composition primarily by eliminating the species that occur in minor abundance (rare species).^26^ ^,^ ^27^

The sandfly species *Ny. antunesi*, *Ps. davisi* and *Th. ubiquitalis* were abundant in all environments. Humans generally come in close proximity to forest fragments while engaged in agriculture or activities like hunting and fishing. These activities increase the risk of exposure to *Leishmania* vectors in possible transmission foci,^25^ ^,^ ^28^ ^)^ and that risk is exacerbated by the abundance of *Ny. antunesi*, *Ps. davisi* and *Th. ubiquitalis* in the areas we studied. One pool of *Ps*. *davisi* tested positive for *Le*. (*Vi*.) *braziliensis* DNA in the municipality of Monte Negro. This finding is significant because *Le*. *braziliensis* is responsible for 50% of human CL cases in the rural population of Monte Negro.^25^ ^,^ ^28^ *Ps. davisi* is an abundant species in this region,^25^ as well as in other parts of RO,^4^ ^,^ ^7^ ^,^ ^13^ and *Ps*. *davisi* individuals have been previously found to be infected with *Le*. (*Vi*.) *braziliensis* ^5^ and *Le*. (*Vi*.) *naiffi.* ^13^ The discovery of this infection in the current study further supports the evidence that *Ps*. *davisi* might act as a vector in RO.

Furthermore, *Ny*. *antunesi* and *Th*. *ubiquitalis* might act as vectors in RO. Both species were found in high abundance in this as well as other studies conducted throughout the state,^4^ ^,^ ^13^ ^,^ ^17^ ^,^ ^25^ and the susceptibility of these species to natural infection by *Leishmania* has been demonstrated in the two studies performed in Porto Velho.^4^ ^,^ ^17^ Furthermore, both species are suspected vectors in AM and MT which border RO.^21^ ^,^ ^29^

Other abundant species found in RO included *Ny*. *whitmani*, *Ps*. *carrerai carrerai*, *Ps*. *complexus*, *Ps*. *hirsutus hirsutus* and *Th*. *auraensis*. *Ny. whitmani* has already been recorded in abundance in RO.^13^ ^,^ ^25^ *Ny*. *whitmani* and *Ps*. *hirsutus hirsutus* were found in high abundance in the FE environments, suggesting that these species are confined to degraded environments; both species have been associated with dense forest environments^13^ ^,^ ^25^ and with environments impacted by anthropic activities.^25^ ^,^ ^30^ Neither species has been found infected with *Leishmania* in RO;^4^ ^,^ ^5^ ^,^ ^7^ ^,^ ^17^ however, both species are putative vectors in the Amazon region because they have been found infected with *Leishmania* in PA.^6^

In RO*, Ps*. *carrerai carrerai* occurs mainly in dense forest environments.^6^ ^,^ ^7^ ^,^ ^13^ Only three studies conducted in central RO have demonstrated the predominance of *Ps*. *carrerai carrerai* and *Ps*. *complexus* in this region.^13^ ^,^ ^25^ *Ps. carrerai carrerai* has been found to carry promastigote flagellates identified as *Le.* (*Vi*.) *braziliensis* ^5^ and carrying *Leishmania* DNA.^7^

*Trichophoromyia auraensis* is abundant primarily in the municipalities of Guajará-Mirim and Porto Velho and in central RO^13^ ^,^ ^17^ where *Leishmania* DNA has been detected in females.^7^ ^,^ ^17^ In our study, *Th. auraensis* was found abundant only in the CUN environments; however, the natural infection of *Th*. *auraensis* by *Leishmania* spp has been reported^30^ and *Th*. *auraensis* has been found in abundance in both FE and PE environments in AC of western Brazil.^19^ ^,^ ^30^

In our analysis of blood meal sources, blood was taken from the stomachs of 15 engorged females, and the PCR amplification of the *cyt*b gene led to the identification of the DNA belonging to humans (*H. sapiens*), domestic animals (*Bos taurus*) and sylvatic animals such as anteaters (*T. tetradactyla*). The DNA belonging to *Bos taurus* and *H. sapiens* was present in samples from every collection made in the PE and FE environments, and this DNA was found in the blood taken from 13 female specimens belonging to the species: *Bi. flaviscutellata*, *Ny*. *antunesi*, *Pa*. *dendrophyla*, *Ps*. *carrerai carrerai*, *Ps*. *davisi* and *Ps*. *hirsutus hirsutus*. The *T. tetradactyla* DNA was present in the blood sample taken from two *Ny*. *antunesi* females captured in the FE environments. Although 15 engorged females represents a small sample size relative to other studies, our findings are significant because previous studies targeting the *cyt*b gene have detected the DNA of only domestic animals, such as cats, dogs, chickens, bovines, equines and pigs.^18^ ^-^ ^20^

Preserved environments showed the highest variety of blood meal sources for sandflies, and a large variety of blood meal sources guarantees the maintenance of the gonotrophic cycle.^12^ Anteaters (*Tamandua* spp) act as possible reservoirs for some *Leishmania* species, such as *Le*. (*Vi*.) *guyanensis*,^12^ and in degraded natural habitats, the scarcity of sylvatic reservoirs, such as anteaters, might cause sandflies to migrate to the PE environments to find new blood meal sources.^4^ ^,^ ^18^ ^,^ ^19^ ^,^ ^27^ In our study, the PE collection points were close to small forest fragments, and the availability of blood meal sources in the form of domestic animals, such as *Bos taurus*, might have attracted sandflies from the forest fragments.

Few studies have examined the importance of blood meal sources in the Brazilian Amazon. Recently, in the municipality of Rio Branco (AC), the blood collected from the intestinal contents of the two specimens of *Ps*. *davisi* was subjected to PCR targeting the *cyt*b gene; this led to the identification of *Gallus gallus* as a blood meal source.^19^ The current study improves our knowledge of blood meal sources by demonstrating that vectors, such as *Ny*. *antunesi* and *Ps*. *davisi*, feed on humans and bovines in the PE environments and feed on sylvatic animals, such as anteaters in the FE environments.

Our study largely corroborates the findings of previous studies concerned with the transmission cycle of leishmaniasis in RO. However, the fact that sandflies are using humans and domestic animals as blood meal sources indicates that the transmission profile might be changing in the PE environments. These findings can be used to enhance the epidemiological surveillance of leishmaniasis in RO.
